# Do the British public recognise differences in survival between three common cancers?

**DOI:** 10.1038/bjc.2012.185

**Published:** 2012-05-03

**Authors:** K L Whitaker, A E Simon, R J Beeken, J Wardle

**Affiliations:** 1 Health Behaviour Research Centre, Department of Epidemiology and Public Health, University College London, Gower Street, London WC1E 6BT, UK

**Keywords:** cancer survival, attitudes, awareness, breast, colorectal, lung

## Abstract

**Background:**

The recognition that cancer is not a single entity, rather that different cancers have different causes and trajectories, has been a key development in the scientific understanding of cancer. However, little is known about the British public’s awareness of differences between cancers. This study examined differences in perceived survivability for three common cancers with widely disparate survival rates (breast, colorectal and lung).

**Method:**

In a population-based survey, using home interviews (*N*=2018), respondents answered a quantitative (numeric) question on 5-year survival and a qualitative (non-numeric) question on curability, for each of the three cancers.

**Results:**

British adults correctly recognised that 5-year survival for breast cancer was higher than for colorectal cancer (CRC), which in turn was recognised to be higher than for lung cancer. Similarly, curability was perceived to be higher for breast than CRC, and both were perceived to be more curable than lung cancer. Awareness of survival differences did not vary by sex, age or socioeconomic status. In terms of absolute values, there was a tendency to underestimate breast cancer survival and overestimate lung cancer survival.

**Conclusion:**

The British public appear to be aware that not all cancers are equally fatal.

The scientific community’s understanding that cancer is not a single entity has been an important theme in oncology over the past century (Mukherjee, 2011). However, popular discourse and media stories often imply a single entity: ‘the war on cancer’, ‘finding a cure for cancer’, ‘one in three of us will get cancer’. There is concern that the general public may not realise that different cancers have very different outcomes. Overestimating cancer survival (equivalent to underestimating its seriousness) may be associated with poor health behaviours such as smoking (Donovan *et al*, 2006). Underestimating survival may be associated with reluctance to participate in early detection. In previous studies, the belief that cancer is a ‘death sentence’ has been found to be associated with negative attitudes towards early detection (Facione and Dodd, 1995; Burgess *et al*, 2001) and with lower screening participation (Chavez *et al*, 1997; Pearlman *et al*, 1999).

Research in the United States indicates that the public are aware of variations in outcome: people rated skin cancer as more survivable than colorectal cancer (CRC), which was rated as more survivable than lung cancer (Rutten *et al*, 2009). Similarly, an Australian study found that breast cancer was perceived as most survivable, followed by CRC, and then lung cancer (Jones *et al*, 2005). However, no similar study has been carried out in Britain despite the fact that cultural differences could limit generalisation across countries. We therefore used data from a British population sample to compare estimated survival for three common cancers distributed across the survival spectrum (breast, colorectal and lung).

## Materials and methods

The survey was incorporated into the BMRB (British Market Research Bureau) omnibus survey in September 2009. The survey uses a random location, quota-sampling technique to ensure that the distribution of respondents (aged ⩾15 years) across area types matches census data; with quota controls set for sex, age, employment status and likelihood of being at home at the time of the interview. A specific person from the household is chosen for interview based on them filling the quota. If the initial person is not available, the person with the next birthday is selected (as an unbiased method of selection). Trained interviewers carried out computer-assisted interviews during household visits. Respondents were questioned about perceived survival and perceived curability for breast, colorectal and lung cancer, except that only women were asked the breast cancer questions. Data were collected from 2018 adults (937 men, 1081 women).

Quantitative survival estimates were based on the question: ‘Out of 100 people with a [breast/colorectal/lung] cancer diagnosis, how many do you think would be alive 5 years later’ with response categories in 10-point intervals from 0–10% through to 91–100%. The qualitative question (perceived curability) was assessed using the statements ‘Many people who get [breast/colorectal/lung] cancer can be completely cured’ with responses on 5-point Likert scales from ‘strongly disagree’ to ‘strongly agree’.

Socioeconomic status (SES) was indexed with the National Readership Survey classification (NRS Ltd, 2007). Group AB comprises people who have (or have had) higher or intermediate managerial or professional occupations, group C1 includes supervisory or junior managerial occupations, group C2 are skilled manual workers, group D are semi- and unskilled manual workers and group E are state pensioners or lowest grade workers. Age and sex were recorded.

### Statistical analysis

Analyses were done using Statistical Package for the Social Sciences (SPSS, Chicago, IL, USA) 17.0 with rim-weighted data (BMRB Target Group Index, 2007). Variables used to define weighting included age, sex, social grade and standard region. We also ran the analyses on unweighted data and there were no significant differences in the results, so only analyses on weighted data are reported. As men did not provide a response for perceived survival/curability of breast cancer, men and women’s responses were analysed separately. Descriptive statistics were conducted for perceived survival and perceived curability. Perceived survival was indexed both with modal scores and by treating it as a continuous scale (from 1 to 10). For perceived survival we also categorised responses for each cancer according to whether they were accurate using CR-UK survival statistics (Cancer Research UK, 2011) into pessimistic (survival estimates lower than actual survival statistics), accurate (survival approximated actual survival) or optimistic (survival estimates higher than actual). Perceived curability was indexed by the proportion of people agreeing or strongly agreeing that ‘many people who get [breast/colorectal/lung] cancer can be completely cured’ and was also scored as a continuous scale from 1 to 5. Repeated measures ANOVAs assessed differences between cancers. Main effects were decomposed using Bonferroni comparisons.

## Results

Respondents were 47.4 years old on average, and 54% were women. Socioeconomic status indicators were well distributed, with 20% in group AB, 27% in C1, 19% in C2, 14% in D and 19% in group E.

Among women, modal 5-year survival estimates indicated that they were aware of differences between cancers, with modal values of 71–80% for breast cancer, 51–60% for CRC and 21–30% for lung cancer. Among men, the modal estimate for CRC (51–60%) was higher than for lung cancer (41–50%); see Table 1.

These survival estimates indicated that as a group, respondents were broadly realistic about breast cancer survival (current 5-year survival is 82%) and CRC survival (current 5-year survival is 50%), but optimistic about lung cancer survival where the current 5-year figure is 8% (Cancer Research UK, 2011).

Categorising individual survival estimates into pessimistic, accurate or optimistic provided directional information about the accuracy of the survival estimates (see Table 2). This revealed a tendency to underestimate breast cancer survival and overestimate lung cancer survival.

The same ranking by cancer site was seen in answers to the qualitative question, where the majority of women (73%) agreed that many people can be completely cured for breast cancer, compared with 48% for CRC, and only 28% for lung cancer. Men were more likely to agree that many people who get CRC can be completely cured (42%) than lung cancer (29%).

Treating responses as continuous variables confirmed significant differences in perceived survival between cancer sites (F(2,1776)=480.92, *P*<0.001). Women perceived breast cancer as significantly more survivable than either of the other cancers (both *P-*values <0.001), and CRC as more survivable than lung cancer (*P*<0.001). There were also significant differences in perceived curability (F(2,2074)=406.71, *P*<0.001), with breast cancer perceived as significantly more curable than CRC (*P*<0.001), which was perceived as more curable than lung cancer (*P*<0.001).

Men’s responses also showed significant differences in perceived survival (F(1,874)=145.86, *P*<0.001) and curability (F(1,967=151.68, *P*<0.001), with CRC seen as significantly more survivable and curable (*P*<0.001) than lung cancer.

Subgroup analyses (lower *vs* higher SES, younger *vs* older) revealed the same pattern of findings (data not shown).

## Discussion

This is the first study to assess perceptions of differences in cancer survival in Britain. It showed that, just like Americans and Australians (Jones *et al*, 2005; Rutten *et al*, 2009), Britons are aware that all cancers are not the same – at least in terms of survival. Estimates of survival and perceived curability varied by cancer type consistent with the rank ordering in Cancer Research UK’s survival statistics (Cancer Research UK, 2011). Breast cancer was perceived to be the most survivable and curable, followed by CRC, and then lung cancer.

Interestingly, although respondents were aware of differences, they nonetheless underestimated survival for the breast cancer and overestimated survival for lung cancer. This may be due partly to the well-established tendency to avoid the extremes on any rating scale, but may also be because although the public know that cancers are different in terms of outcomes, they do not realise quite how different.

Although average (modal) survival estimates were correctly ranked, there was considerable variability in survival estimates at the individual level; indicating a role for public education about cancer outcomes. While reducing fatalistic beliefs about breast cancer survival may increase early detection behaviours, such as screening participation and promote timely presentation, public health campaigns aimed at encouraging people to stop smoking may want to place more emphasis on the high mortality rates of lung cancer.

The measures we used had advantages and disadvantages. The use of numerical ratings for survival has the advantage of making it possible to compare the values with ‘true’ survival, but the cost is higher frequency of missing data than for the ‘qualitative’ question (i.e., more people answered the curability than the survival question). This was particularly evident for CRC, which may be because of lower awareness of CRC than other common cancers (Juszczyk *et al*, 2011). The amount of missing data did not vary by sex or SES, but older respondents (>65 years) were less likely to answer the numeric question. Lay people may have difficulty understanding numeric estimates of risk (Reyna *et al*, 2009) and this needs to be taken into account in assessing public awareness.

In common with previous research, we used single-item scales to measure perceptions of survival and cure, which are less reliable, but allowed the results to be compared with previous findings (Jones *et al*, 2005; Rutten *et al*, 2009). We found a ‘floor effect’ in the scale for lung cancer survival because of using categories covering 10 percentage points, which meant that the lowest category (1–10%) contained the true survival figure. However, only 11% of respondents chose the lowest category. Finally, the fact that only women were asked the breast cancer questions limited the analyses in men to two cancers. The results showed that men were significantly more optimistic about lung cancer survival than women, although reasons for this are unclear.

Notwithstanding these caveats, these results demonstrate clearly that concern that the public are unaware that different cancers have different outcomes is unfounded. Men and women, from all age and SES groups, have evidently learned that some cancers have better outcomes and others are more likely to be fatal. Future public information about cancer can build on this foundation.

## Figures and Tables

**Table 1:** Perceived survival and curability by cancer site for women and men (weighted data)

**Cancer site by sex**	**Modal survival estimate (%)**	**% Agreeing that many people who get [..]cancers can be completely cured**	**Perceived survival (scored 1**–**10), mean (s.d.)**	**Perceived curability (scored 1**–**5), mean (s.d.)**
*Women* (*n=1038*^a^)				
Breast	71–80	73	6.61 (1.96)^b^^1^	3.85 (0.96)^b^^1^
CRC	51–60	48	5.53 (2.08)^2^	3.44 (0.96)^2^
Lung	21–30	26	4.41 (2.01)^3^	2.89 (0.93)^3^

*Men* (*n=980*^a^)				
CRC	51–60	42	5.32 (2.09)^1^	3.34 (0.90)^1^
Lung	41–50	29	4.44 (2.12)^2^	2.93 (0.99)^2^

Means followed by different numbered superscripts differ significantly (*P*<0.001).

a *n*’s appear different because analyses are done on weighted data.

b Missing data range from *n*=70 (breast cancer) to *n*=203 (colorectal cancer, CRC) for survival estimates and *n*=2 (breast cancer) to *n*=3 (CRC, lung) for curability estimates. Missing data includes ‘refused’, ‘don’t know’ and ‘not stated’ options.

**Table 2:** Frequency data showing perceived 5-year survival by cancer site for men and women (weighted data)

**Cancer site by sex**	**Pessimistic % (*n*)**	**Accurate % (*n*)**	**Optimistic % (*n*)**
*Women (n=1038*^a^)			
Breast	60 (581)^b^	35.3 (342)	4.7 (45)
CRC	29.7 (273)	39.8 (367)	30.5 (281)
Lung	___	9.6 (90)	90.4 (842)
			
*Men (n=980*^a^)			
CRC	30.5 (272)	41.4 (371)	28.1 (251)
Lung	___	11.8 (109)	88.2 (811)

a *n*’s appear different because analyses are done on weighted data.

b Missing data for survival estimates, which range from *n*=70 (breast cancer) to *n*=203 (colorectal cancer, CRC), include ‘refused’, ‘don’t know’ and ‘not stated’ options.
